# The Wisconsin Medical Association

**Published:** 1853-08

**Authors:** 


*The Wisconsin Medical Association.*

The following named gentlemen, being present at Janesville on Wednesday, the 15th of June ult., to attend the meeting of the State Medical Society, and finding themselves unable—except Messrs. Lathrop, Castleman and Wilber, permanent members—to become members thereof, or to participate in the doings of the Society, agreed to associate themselves together to form a new State Medical Society.

Gentlemen present: Prof. L. P. Lathrop, and Dr. C. J. Taggart, of Beloit; Dr. D. Cooper Ayres, of Green Bay; Dr. Peter Winters, of Horricane; Dr. Alfred L. Castleman, of Delafield;

Dr. R. T. Hayes, of Platteville; Dr. Geo. D. Wilber, of Mineral Point; Dr. J. P. Hamilton, of Highland; Dr. J. Fayville, of Madison; and Drs. L. J. Barrows, C. G. Pease, and C. C. Bradly, of Janesville.

The meeting was organised by calling Prof. Lathrop to the Chair, and appointing Dr. Wilber Secretary.

The preliminary steps were taken for the organization of a Society to be known as "The Wisconsin Medical Association"; and Drs. Castleman, Fayville, and Taggart, were appointed to report at the next meeting, a Constitution and By-Laws for the permanent organization of the Association.

The following are the Officers of the Association until its permanent organization, and were elected by ballot, namely :—Dr. Castleman, President; Dr. Ayres, Vice Prasident; Dr. Wilber, Recording Secretary; Dr. Pease, Corresponding Secretary; Dr. Hayes, Treasurer; Prof. Lathrop, Dr. Barrows, and Dr. Taggart, Censors.

Dr. Ayres was appointed to deliver a public address at the next meeting of the association, on the subject of Lunacy and the importance of the immediate establishment of a State Lunatic Asylum in Wisconsin.

After the transaction of some other business, the Association adjourned to meet at Madison on the fourth Wednesday of January A.D. 1854, at 12 o'clock, M. All regular Physicians throughout Wisconsin, are particularly requested to bear the meeting in mind, and, if possible, be in attendance.

Atttest

ALFRED L. CASTLEMAN,
*President.*

